# Cognitive Function and Delayed Medical Care During Covid-19: Does Family Social Support Moderate This Association?

**DOI:** 10.1093/geroni/igaf122.2306

**Published:** 2025-12-31

**Authors:** Brittany McFeeley

**Affiliations:** University of Massachusetts Boston, Boston, Massachusetts, United States

## Abstract

During the pandemic, healthcare facilities postponed or cancelled elective procedures and patients delayed their care to avoid the infection. Little is known about the role of cognitive function for older adults who delayed care. Further, social support from spouses and children may have protected against delays in medical care during the pandemic. This study used the 2020 wave of the Health and Retirement Study (N = 4,087) to examine the relationship between cognitive function and delaying medical care and whether positive or negative social support from family had a moderating effect. Cognitive function was measured with the Telephone Interview for Cognitive Status and delaying medical care was a binary variable (1=delayed care). Positive and negative support scales from spouses and children were converted to z-scores. Results showed that 30% of older adults reported delaying their medical care. Those with better cognitive function (OR = 1.03, 95%CI=1.01-1.05, p=.001) and those who reported more negative support from children (OR = 1.14, 95%CI=1.05-1.24, p=.002) were more likely to delay care. The moderation results were not statistically significant. Older adults with better cognitive function may have better understood the social distancing guidelines and threats of Covid-19. Older adults who felt unsupported by their children may be less likely to share health concerns or seek care. Future research should examine whether specific domains of cognitive function are related to seeking medical care during public health crises.

